# Finding functionality: Rasch analysis of the Functionality Appreciation Scale in community-dwelling adults in the US

**DOI:** 10.3389/fresc.2023.1222892

**Published:** 2023-10-02

**Authors:** Sarah Feng, Sydney McDaniel, Ann Van de Winckel

**Affiliations:** ^1^ Breck School, Golden Valley, MN, United States; ^2^ Division of Physical Therapy, Division of Rehabilitation Science, Department of Rehabilitation Medicine, Medical School, University of Minnesota, Minneapolis, MN, United States

**Keywords:** body image, validation studies, functionality, appreciation, spinal cord injury, body awareness

## Abstract

**Introduction:**

The Functionality Appreciation Scale (FAS) measures an individual's appreciation for the functions their body can perform, regardless of the individual's physical limitations. Prior studies reported on internal consistency, test-retest reliability, convergent validity, and exploratory or confirmatory factor analyses, but Rasch analysis has not yet been performed to evaluate the structural validity of the FAS.

**Methods:**

We recruited community-dwelling adults at the Minnesota State Fair and through contact lists of participants identifying interest in research done in the Brain Body Mind Lab (University of Minnesota). Community-dwelling adults with spinal cord injury (SCI) completed the FAS over Zoom. We analyzed the FAS using Rasch Measurement Theory, which produced the following outputs: item, and person fit, targeting, unidimensionality, person separation reliability (PSR), local item dependence (LID), principal component analysis of residuals (PCAR), and differential item functioning (DIF).

**Results:**

We recruited 567 participants (average age 52.15 ± 17.5 years, 63.84% women), among which 14 adults with SCI. After rescoring 3 items and deleting 1 item, the FAS had good person and item fit (except item 4). The PCAR and subsequent paired *t*-tests (3.53%) confirmed the unidimensionality of the scale. There was no DIF and only one item pair had LID (item 5–6). PSR was 0.75, reflecting a capacity to differentiate groups of people with high or low functionality appreciation levels. However, there was a significant ceiling effect (28.04%) and the person mean location was 3.06 ± 2.07 logits, indicating the FAS is too easy for community-dwelling adults in the US.

**Discussion:**

The 6-item Rasch-based FAS demonstrated unidimensionality, good item fit (except item 4) and person fit, but the FAS will require more difficult items to be added to improve the targeting of the scale, and better reliability.

## 1. Introduction

Functionality appreciation is characterized by the acknowledgment and value of everything the body is capable of doing, from communication to physical capacities (1). As research on the effects of mindfulness and other mind and body approaches are increasingly being used for treating chronic pain, mental health disorders, rehabilitation from injury, and other health conditions, it has become clear that body awareness and body image play a role in overall physical and mental well-being (2). The appreciation of body functionality has also shown a positive correlation with improved body image, establishing itself as a major dimension of the construct of body image (3).

Previous studies on body functionality have sought to define the concept under limited terms of which bodily systems may qualify as “functional.” However, there are various forms of body functionality, specifically relating to internal functions and external functions. Some internal examples include digestion, the senses of sight or smell, and creativity. External functions include interaction with others, physical capabilities in movement, and hygienic practices (1). In a more inclusive sense, body functionality acknowledges the way the body functions according to its ability to accomplish specific needs, as opposed to limiting the definition of functionality to carrying out these processes in a particular way (1).

Most researchers have assessed functionality appreciation with surveys rather than physical activity, as it is more a psychological concept than a physiological one. The first generation of questionnaires assessing body functionality pertained only to specific domains of body functionality. For example, the Physical Condition Subscale of the Body Esteem Scale included questions pertaining mainly to the physicality and sexualization of the body (4). The Self Objectification Questionnaire focused on the physical objectification of women relative to physicality (5). The Body Surveillance Subscale of the Objectified Body Consciousness Scale again targets body shame and appearance control in women, ignoring other groups affected by the constructs (6). These scales, although making important contributions to the research of body functionality, do not capture body functionality in a holistic manner that goes beyond physical appearance and abilities.

The Functionality Appreciation Scale (FAS) has become the most widely used scale for the measure of body functionality (3). Consisting of seven items, the FAS was designed to measure body functionality appreciation holistically, i.e., not specific to any one domain of body functionality (3). Thus, items assess not only physical capability, but also internal processes, bodily perceptions, creative endeavors, and communication with others (1). Participants score the seven items on a range from 0 (strongly disagree) to 4 (strongly agree). An example of such an item is “*I appreciate my body for what it is capable of doing*.” Items are also designed to be all-inclusive regarding physical capacity, thereby including adults with physical disabilities (3).

Alleva & Tylka (1) demonstrated that FAS has good internal consistency, test-retest reliability, is correlated with aspects of positive body image and well-being (e.g., body appreciation, self-esteem, self-compassion), and inversely correlated with aspects of negative body image and ill-being (e.g., self-objectification, depression) (1). Since scales that measure body awareness and body image can be used as outcome measures to evaluate the effectiveness of an intervention, it is imperative to investigate structural validity, i.e., evaluating whether the items on the scale and the scale as a whole are measuring the construct they are meant to measure (7). Within this context, unidimensionality pertains to whether a scale is assessing a single construct or trait. Exploratory and confirmatory factor analyses have been conducted in the US, Europe, Asia, and Australia (3, 8–13). Lindardon et al. (8) identified sex invariance through confirmatory factor analysis (8). Validated translations of the FAS are available in Farsi, Italian, Japanese, Malay, and Romanian (9–13).

However, no previous study has conducted a Rasch analysis on the FAS. Rasch Measurement Theory evaluates structural validity through a probability model that states that if a person has a higher ability on a certain trait (e.g., functional appreciation; motor function), that person should have a greater probability of obtaining a higher score (14). Rasch analysis examines the structural validity, including unidimensionality, of the scale, group invariance, and orders the items hierarchy from easy to difficult (14). Rasch analysis also converts the original ordinal scale into an interval scale where requirements of the model are met, providing thereby a more precise measurement in the clinic and in research (14). It places the ability of participants and the difficulty of items on one continuous ruler, using “logits” as a unit of measurement (14, 15). The aim of the present study is to determine the structural validity of the FAS in community-dwelling adults in the US using Rasch Measurement Theory.

## 2. Materials and methods

### 2.1. Participants

Participants were recruited at the Minnesota State Fair and Highland Fest. We included English-speaking adults (18+). We excluded women who were pregnant because the pregnancy could temporarily bias the appreciation of the functionality of their bodies. General demographic, general health, and lifestyle information were collected prior to the FAS assessment on an iPad. Participants were asked whether they had ever done or were currently performing breathing exercises, mindfulness or relaxation exercises, or other body awareness practices such as Qigong, Tai Chi, Yoga, or martial arts.

The Institutional Review Board of the University of Minnesota approved the study (IRB# STUDY00005849). The study was performed in accordance with the Declaration of Helsinki (16). Since this was an anonymous survey where no identifying information was collected, consent was not signed but was acknowledged through the provided forms. Additionally, participants completed the University of California, San Diego Brief Assessment of Capacity to Consent as proof of their understanding of the consent (17). Participants unable to obtain a perfect score of 20 on the Assessment were excluded from the study. Healthy participants who had expressed interest in participating in research from the Brain Body Mind Lab were invited to participate in this research project through an e-mail with a link to the questionnaire. Since no identifying information was collected, it was not possible to trace who responded to this request.

Baseline FAS results were also collected from a group of community-dwelling adults with spinal cord injury (IRB# STUDY00008476) over the University of Minnesota's secure Zoom platform. They signed informed HIPAA/eConsent through the secure REDCap platform of the University of Minnesota and participated in a clinical trial study for reduction of neuropathic pain (18). Only baseline assessments were used for the Rasch analysis.

### 2.2. Outcome measure

The FAS has 7 items, ranging from 0 (strongly disagree) to 4 (strongly agree) with higher scores reflecting a higher level of appreciation for the functionality of the body. Thus, the scale has a range of 0–28 where a high score reflects better body functionality appreciation.

### 2.3. Statistical analysis: Rasch analysis

Rasch Measurement Theory evaluates structural validity: whether the scale is measuring one dimension (unidimensionality) and whether the items and the scale as a whole are fitting the Rasch model. Rasch analysis was performed with the Rasch Unidimensional Measurement 2030 Software (RUMM2030) (19). We followed the Rasch Reporting Guideline for Rehabilitation Research (RULER) to report our results (20, 21).

The Rasch analysis produces several outcomes:

(1) It verifies whether scoring categories for each item are fitting the probabilistic model, and alerts when scoring categories show **reversed thresholds**, usually requiring scoring categories to be merged (i.e., rescoring of the items) (22).
(2) **Overall fit, item fit, and person fit** are analyzed with Chi-Square statistics to verify whether the observed scores match the expected scores of the probability model. Residuals greater than 2.5 with a significant *p*-value indicate item or person misfit (7).
(3) The **person separation reliability (PSR)** outcome measures how well we can differentiate high abilities from low abilities in a specific trait in persons (23). PSR ranges from 0.00 to 1.00, where a higher PSR indicates a better separation and a more precise measurement (24). A score above 0.70 allows us to distinguish different abilities in groups; a score above 0.9 allows us to distinguish levels of ability in individual persons (20). Note that, in RUMM 2030, this output is called the Person Separation Index (20). The **mean error variance** is a type of standard error of measurement (25).
(4) Good **targeting** is obtained when the average person location (in logits) is within a range of −0.5 to +0.5 logits of the average item location, which by default is set at 0 logits (26). Moreover, floor and ceiling effects need to be reported when 15% or more participants have a minimum or maximum score on the scale (27).
(5) **Differential item functioning (DIF)** evaluates whether the hierarchy of item difficulty is maintained across demographic, clinical, or behavioral variables and can be calculated when subgroups have a sample size of *n* = 200 or greater (20). DIF occurs when the responses from one group are shifted more than 0.5 logits from the other group. The variables for which we investigated DIF were sex (male, female, other); currently doing breathing exercises (yes, no); currently doing body awareness training (yes, no).
(6) **Principal component analysis of residuals (PCAR)** is used to investigate unidimensionality by extracting the common factor that explains the most residual variance under the hypothesis that there is such a factor. Ideally, the percentage of total variance accounted for by the first principal component should be less than 10% with an eigenvalue of less than 2. The latter reflects that the variance is explained by 1 underlying trait. If this is not the case, then paired *t*-tests can be used between 2 subtests of items that load positively and negatively (with correlations smaller than −0.3 or larger than 0.3) on the first principal component, to investigate unidimensionality further. We can assume unidimensionality of the scale if those paired *t*-tests report less than 5% significant differences in person locations on the two subtests (7).
(7) Residual correlations reflect a degree of **local item dependence (LID)**. This test examines whether two items have more in common with each other than with the whole scale. LID is reported when two items have a correlation of at least 0.2 above the average residual item correlation (28).

Bonferroni correction was applied for all statistical analyses that involved multiple comparisons.

## 3. Results

### 3.1. Demographic and behavioral data

We recruited 567 community-dwelling adults between June 2019 and September 2021, who were on average 52.15 ± 17.50 years old, 64% women, 89% were White, 40% was doing breathing exercises, and 32% was doing body awareness training. Among this sample, we recruited 14 adults with spinal cord injury (SCI)-related neuropathic pain, who were 1–45 years post-SCI, with a spine lesion at locations between the C4 and L1 vertebrae.

**Figure 1:**
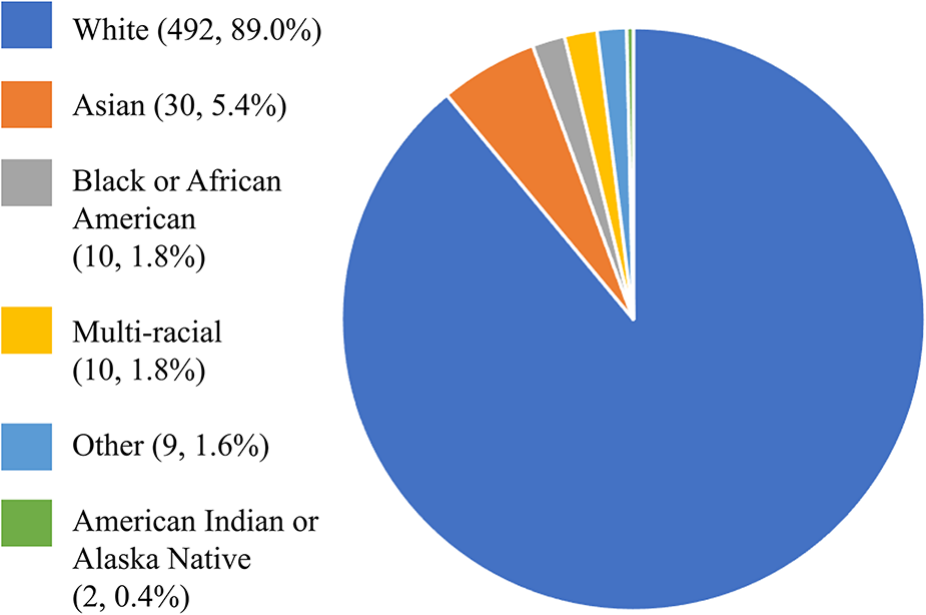
Race distribution. The pie chart shows the race distribution of the 567 participants, demonstrating the lack of diversity in the group of community-dwelling adults tested in Minnesota.

More details on the demographic, general health, and lifestyle data are presented in Table 1. Figure 1 illustrates the race distribution of the participants.

**Table 1:** Demographic, general health, and lifestyle characteristics of the participants.

	Total participants (*n* = 567)
Age (years, mean ± SD)	52.15 ± 17.50
65 years old or above (*n*, %)	162 (28.57)
Less than 65 years old (*n*, %)	405 (71.43)
Sex (*n*, %)	
Women	362 (63.84)
Men	204 (35.98)
Other	1 (0.18)
Self-reported mental health conditions (*n*, %)	193 (34.04)
Self-reported pain (*n*, %)	136 (23.99)
Spinal cord injury (*n*, %)	14 (2.47)
Ever done breathing exercises (*n*, %)	351 (61.90)
Currently doing breathing exercises (*n*, %)	231 (40.74)
Ever done mindfulness or relaxation exercises (*n*, %)	279^a^ (50.45)
Current mindfulness or relaxation exercises (*n*, %)	181^a^ (32.73)
Ever done body awareness training (*n*, %)	359 (63.32)
Currently doing body awareness training (*n*, %)	183 (32.28)

^a^ Data not assessed in adults with spinal cord injury. Body awareness training included but was not limited to dance training, martial arts, Tai Chi, Qigong, Yoga, Pilates, barre, and P. Volve exercises (i.e., a fitness program developed in New York).

### 3.2. Rasch Measurement Theory analysis

The iteration analysis (Table 2) displays the results of each step of the Rasch analysis. The main results are presented below.

**Table 2:** Iteration analysis table of the FAS.

Analysis	Items	Rating scale catego-ries	Person Mean (SD) logits	Mean error va-riance	Floor effect *n* (%)	Ceiling effect *n* (%)	Overall *χ*^2^ (DF) *p*-value	PSR	Items with disordered thresholds	Mis-fitting Items	PCAR Eigenvalue 1st contrast *n* (%)	Mis-fitting persons *n* (%)
All items (*n* = 567)	7	35	3.64 (2.09)	0.91	0 (0.00%)	153 (26.98%)	83.95 (42) *p *= 0.00013	0.78	2 (items 1,4)	1 (item 7)	1.73 (24.78%) paired *t*-test: 3.35%	2 (0.35%)
Rescore items 1,4 to 0 0 1 2 3 (*n* = 567)	7	33	3.54 (2.10)	0.91	0.00%	153 (26.58%)	81.21 (42) *p *= 0.00027	0.79	0	1 (item 7)	1.73 (24.72%) paired *t*-test: 3.35%	2 (0.35%)
Rescore item 7 to 0 0 1 2 3 (*n* = 567)	7	32	3.28 (2.10)	0.90	0.00%	153 (26.58%)	81.23 (42) *p = *0.00027	0.79	0	1 (item 7)	1.73 (24.72%) paired *t*-test: 3.35%	2 (0.35%)
Delete item 3 (*n* = 567)	6	27	3.06 (2.07)	0.99	0.00%	159 (28.04%)	81.38 (30) *p < *0.001	0.75	0	1 (item 7)	1.68 (28.02%) paired *t*-test: 3.53%	3 (0.53%)

DF, degrees of freedom; PCAR, principal components analysis of residuals; PSR, person separation reliability; SD, standard deviation.

Two items were rescored because they had reversed thresholds. Original items numbers 1 (“*I appreciate my body for what it is capable of doing*”) and 4 (“*I acknowledge and appreciate when my body feels good and/or relaxed*”) were rescored from [0 1 2 3 4] to [0 0 1 2 3]. Next, original item number 7 (“*I respect my body for the functions that it performs*”) did not fit the model (Residual = −5.83, *p *= 0.001) and was first rescored from [0 1 2 3 4] to [0 0 1 2 3]. A visual observation of the person-item threshold distribution showed that the lowest scoring category threshold (between score 0 and 1) of original item number 3 was at an extreme left position of the scale, creating an artificially large logit ruler range (from −6.5 to 5.6 logits). After deleting item 3, the logit range was from −3.4 to 5.6 logits. Original item number 7 still displayed misfit (Residual = −5.95, *p < *0.001), but deleting item 7 reduced the PSR to unacceptable levels (PSR = 0.67). Therefore, original item 7 (now item 4 in the Rasch-based FAS) was kept in. The remaining 6 item locations (listed from easiest to hardest item, top to bottom) and Chi-Square statistics reflecting item fit are displayed in Table 3. The scale demonstrated excellent person fit, as only 0.53% (3 participants out of 567) had a fit residual greater than 2.5.

**Table 3:** Item fit statistics of the Rasch-based FAS.

Item number	Item difficulty (Logits)	SE	χ^2^	Item Fit Residual	*p*-value
1	−0.67	0.09	11.73	−3.511	0.04
2	−0.37	0.09	9.39	−0.58	0.09
3	−0.31	0.09	8.19	−2.34	0.17
4	0.10	0.09	27.19	−5.95	<0.001
5	0.62	0.10	11.49	0.13	0.04
6	0.63	0.09	13.40	2.30	0.02

SE, standard error; *p*-values are Bonferroni-corrected for multiple comparisons (*α* corrected value at 0.008).

**Figure 2:**
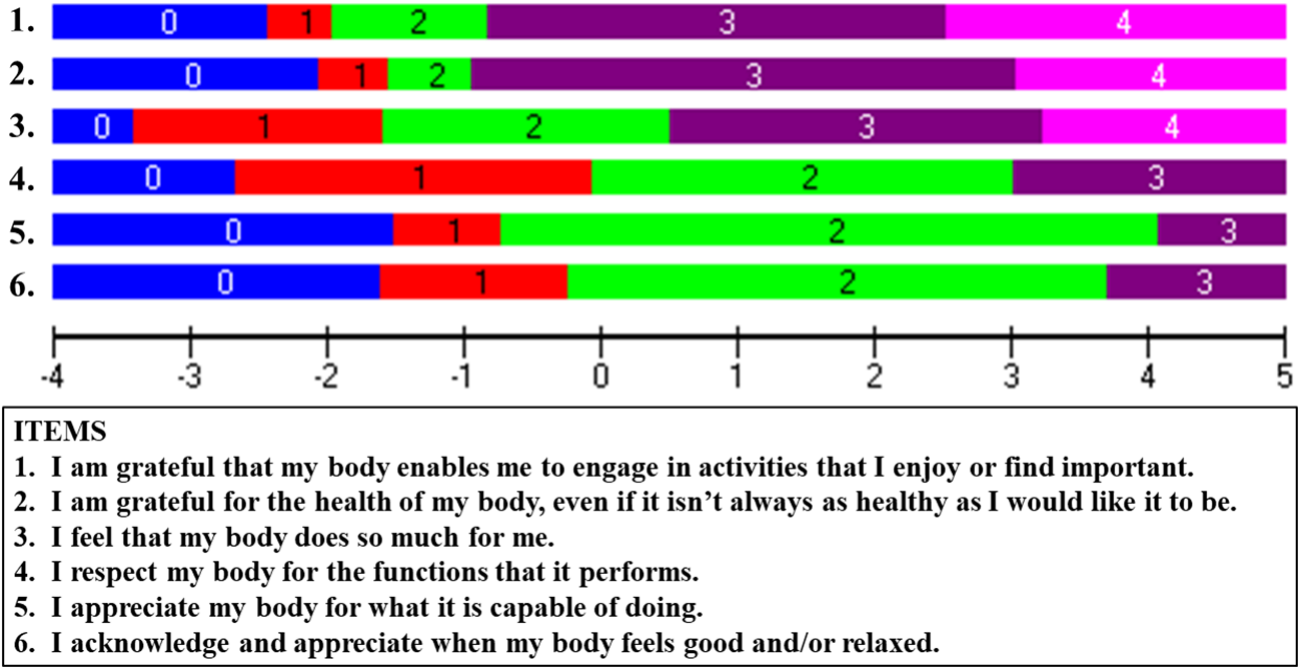
Item threshold Map. The item threshold map shows items arranged in order of difficulty (top to bottom) from easiest (“*I am grateful that my body enables me to engage in activities that I enjoy or find important*”) to hardest (“*I acknowledge and appreciate when my body feels good and/or relaxed*”). The horizontal ruler indicates the logits spanning the item threshold difficulties.

Table 4 shows the Rasch-based scoring sheet after rescoring original items numbers 1, 4 and 7, and removing original item number 3. The items were renumbered to reflect the hierarchical order from the easiest item, “*I am grateful that my body enables me to engage in activities that I enjoy or find important*”, at the top, to the most difficult item, “*I acknowledge and appreciate when my body feels good and/or relaxed.*”, at the bottom. The item threshold map shows similar information in a visual representation (easiest to hardest; top to bottom) along a logit scale ranging from −3.4 to 5.6 logits with the scoring category thresholds for each item displayed in Figure 2.

**Table 4:** Scoring form of the Rasch-based FAS with rescored items.

Items (easy to hard)	Strongly disagree	Disagree	Neither agree nor disagree	Agree	Strongly agree
	0	1	2	3	4
1. I am grateful that my body enables me to engage in activities that I enjoy or find important.	0	1	2	3	4
2. I am grateful for the health of my body, even if it isn't always as healthy as I would like it to be.	0	1	2	3	4
3. I feel that my body does so much for me.	0	1	2	3	4
4. I respect my body for the functions that it performs.	0	0	1	2	3
5. I appreciate my body for what it is capable of doing.	0	0	1	2	3
6. I acknowledge and appreciate when my body feels good and/or relaxed.	0	0	1	2	3

**Figure 3:**
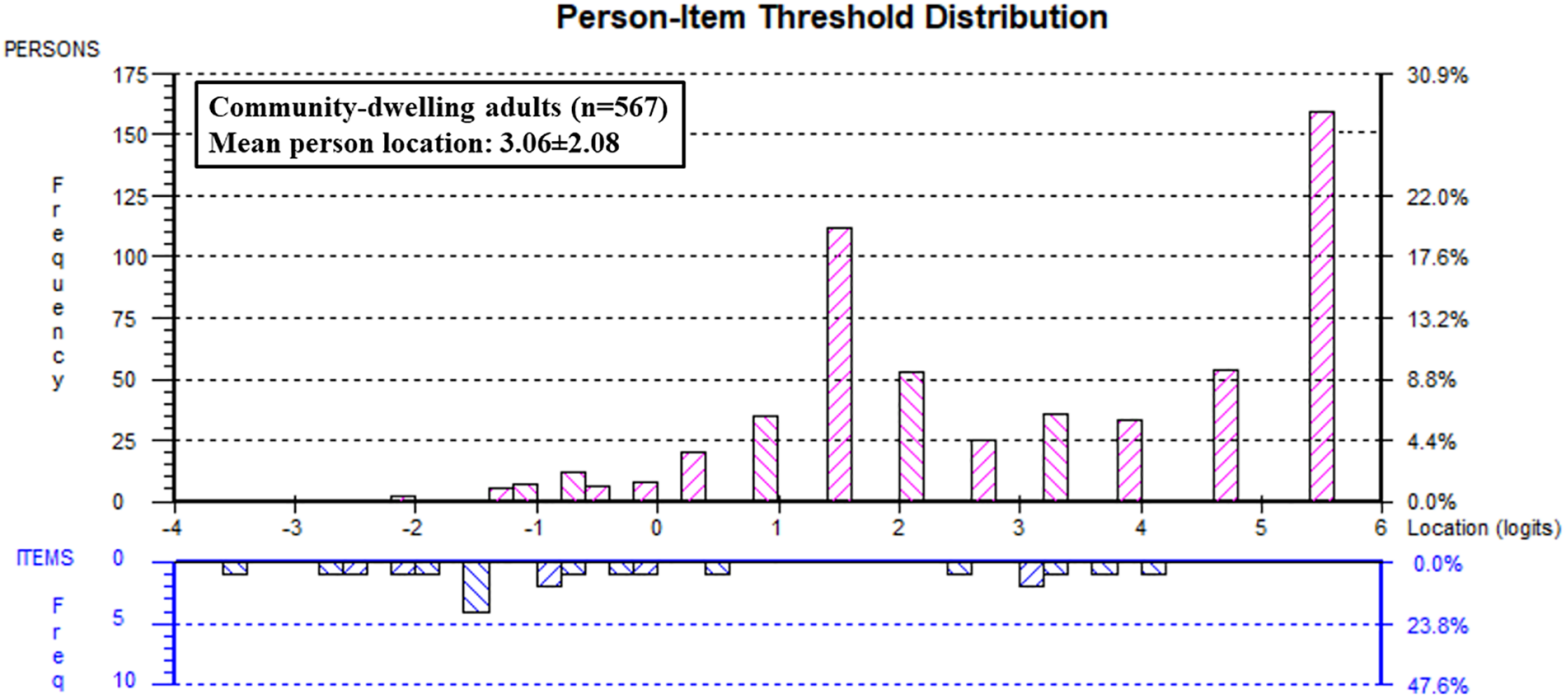
Person-item threshold distribution. The person-item threshold distribution contains histograms that indicate the frequency of participants at different functionality appreciation ability levels (logit scores). The histograms are split and organized by frequency of person ability levels at the top (pink diagonal lines), organized from having a low (left side) to a high functionality appreciation (right side of the graph). The histogram below (blue diagonal lines) indicates the frequency of item thresholds organized along the same logit scale (from easiest items on the left to hardest on the right side of the ruler).

The PSR value for the FAS was 0.75, which means that it is possible to differentiate two groups of people according to their ability level of functionality appreciation (20). There was no floor effect (0.00%), but there was a significant ceiling effect with 159 participants out of 567 obtaining a maximum score (28.04%). The person mean location was 3.06 ± 2.07 logits, meaning that the items were too easy for community-dwelling adults in the US (Figure 3). Table 5 provides the transformation table from the ordinal scores to logits to conversion from logits to a 0–100 scale.

**Table 5:** Conversion table.

Total FAS score	Logit conversion	Converted logits to 0–100
0	−4.70	0.00
1	−3.79	8.80
2	−3.16	14.92
3	−2.72	19.19
4	−2.38	22.54
5	−2.08	25.41
6	−1.81	28.03
7	−1.56	30.52
8	−1.31	32.98
9	−1.05	35.50
10	−0.77	38.21
11	−0.46	41.18
12	−0.11	44.60
13	0.31	48.67
14	0.82	53.70
15	1.46	59.90
16	2.13	66.40
17	2.74	72.31
18	3.31	77.93
19	3.92	83.82
20	4.67	91.08
21	5.58	100.00

The PCAR eigenvalue was 1.68 with a percent variance of 28.02%. The paired *t*-tests revealed that only 3.53% of persons had significantly different person locations among the two subsets of items, thereby supporting the unidimensionality of this scale for measuring functionality appreciation.

There was no DIF found for any of our variables. Consequently, item difficulty was working in the same way regardless of sex, or whether participants performed breathing or body awareness training. LID was found for (originally numbered) items 5 and 6 (*r *= 0.19). In the new Rasch-based FAS, those are items 1 “*I am grateful that my body enables me to engage in activities that I enjoy or find important*” and 3 “*I feel that my body does so much for me*” (Supplementary Table S1).

### 3.3. Descriptive statistics of the subgroups as identified by DIF

The person mean locations of the subgroups of sex, doing current breathing or body awareness exercises are displayed in Supplementary Table S2. Given that there was no DIF, we performed *t*-tests on the person locations to identify whether any differences emerged among those subgroups. There were no significant differences between the subgroups of sex, or whether they currently do breathing exercises. The subgroup that currently does body awareness training in daily life scored higher on FAS than the group that does not currently do any body awareness training (*p < *0.0001).

## 4. Discussion

Functionality appreciation represents an important dimension in improving overall body image that is not based on physical appearance (3). Our Rasch analysis demonstrated that the Rasch-based modified FAS has good structural validity reflected by good item and person fit, once 3 items were rescored (by collapsing categories) and 1 item was removed. However, in this Rasch-based FAS, one item still displays misfit (item 4) but removing it further decreased the reliability, so we decided to keep this item in. The Rasch-based FAS now has 6 items, with 3 items scoring 0–3 and 3 items scoring 0–4. Even though the overall *χ*^2^ (Table 2) was still significant for the 6-item Rasch-based FAS, unidimensionality was confirmed through the paired *t*-tests. Unidimensionality was also identified through exploratory or confirmatory factor analyses in previous studies (3, 9–13). The reason for LID in item pair 1–3 is not straightforward but may be related to the appreciation for what the body is “doing” (activity, tasks,…), whereas other items may reflect more the gratitude towards the “state” of the body (health, functioning, capable, feels good, etc.).

With the exception of Sahlan et al. (12) who tested the FAS in adolescents, all other studies assessed the FAS in adults, in a non-clinical setting (3, 9–13). An important identified problem with Rasch analysis was the significant ceiling effect (28.04%), also reflected by the person mean location, which revealed that the items were too easy for community-dwelling adults. Items with a higher difficulty level (i.e., indicating a more challenging aspect of body functionality) would need to be added and validity retested to improve the targeting of this scale for use in community-dwelling adults. Suggestions for more difficult items to be tested could be “I am grateful that I can keep breathing and talking calmly when I have a difficult conversation with someone” or “I appreciate that I can remain calm and think clearly when I am in a stressful situation”. In addition to the ceiling effect, there is a substantial group of community-dwelling adults at about 1.5 logits (Figure 3) where there is an absence of item thresholds, reducing their precision of measurement.

On the other hand, if the FAS is meant to be used in clinical settings, for prevention and treatment of negative body image and in the enhancement of positive body image (3), then further Rasch analyses should be focused on these clinical populations (e.g., adults or adolescents with eating disorders; adults with physical impairments such as adults with SCI; or adults with severe injuries such as severe burns or scars), and not in community-dwelling adults. Our results could then serve as a normative sample (i.e., what score range can be expected in a sample without apparent body functionality appreciation problems).

Two limitations to this study are worth noting. First, our sample lacks diversity. The State of Minnesota has an 81.64% White population and a 6.43% Black or African American population (29), which explains why our sample consisted of 89.0% White participants, and only 11% of other races, including 5.4% Asian, 1.8% Black or African American, 1.8% multiracial, 1.6% other, and 0.4% American Indian or Alaska Native participants. Even though, to date, the FAS has been translated in at least 5 languages and FAS psychometrics have been investigated across 4 continents encompassing different cultures, more work needs to be done to evaluate the FAS in more diverse and inclusive groups, keeping in mind the DIF requirements of *n* = 200 per subgroup. Second, our sample of community-dwelling participants included 14 adults with SCI and neuropathic pain. It would be worthwhile investigating the structural validity of the FAS in a larger sample size of adults with SCI, adults with other neurological disorders, or adults with chronic pain.

In conclusion, the 6-item Rasch-based FAS demonstrates good item fit (except item 4) and person fit, and unidimensionality through the paired *t*-test. Specifically for use of the Rasch-based FAS in community-dwelling adults, reliability needs to be improved, and targeting needs to be addressed. If the FAS is meant to be used in a clinical population, then further Rasch analyses in such populations are warranted.

## Data Availability

The datasets analyzed for this study can be found in the Dryad repository: https://doi.org/10.5061/dryad.6t1g1jx43.
