# Supplementary material for: Finding functionality: Rasch analysis of the Functionality Appreciation Scale in community-dwelling adults in the US
Source: Front Rehabil Sci. 2023 Oct 2;4:1222892. doi: 10.3389/fresc.2023.1222892 (PMC10577199; doi:10.3389/fresc.2023.1222892)
Supplement: Supplementary file 1 [file Table1.docx]

**Supplementary Table 1**. Residual correlations

| ITEMS | 1 | 2 | 3 | 4 | 5 | 6 |
| --- | --- | --- | --- | --- | --- | --- |
| 1 |  |  |  |  |  |  |
| 2 | -0.059 |  |  |  |  |  |
| 3 | -0.198 | -0.248 |  |  |  |  |
| 4 | -0.264 | -0.068 | -0.160 |  |  |  |
| 5 | -0.267 | -0.316 | -0.367 | -0.179 |  |  |
| 6 | -0.251 | -0.297 | -0.214 | -0.169 | **0.186** |  |

Average *r*= -0.1914

LID would be identified for any item pair with a value above *r*=0.0086

**Supplementary Table 2**. Person mean locations organized by subgroup as identified by DIF

| DIF subgroups | n, Person mean location subgroup 1 | n, Person mean location subgroup 2 | Mann-Whitney U test |
| --- | --- | --- | --- |
| Sex (male, female) | n=204  3.02 ± 2.01 | n=362  3.08 ± 2.11 | Z=-0.45  *p*=0.65 |
| Currently doing breathing exercises (yes, no) | n=231  3.25 ± 2.11 | n=336  2.94 ± 2.05 | Z=1.98  *p*=0.05 |
| Currently doing body awareness training (yes, no) | n=183  3.63 ± 1.93 | n=384  2.79 ± 2.09 | Z=4.42  *p<*0.0001 |
